# General and specific responsiveness of the amygdala during explicit emotion recognition in females and males

**DOI:** 10.1186/1471-2202-10-91

**Published:** 2009-08-04

**Authors:** Birgit Derntl, Ute Habel, Christian Windischberger, Simon Robinson, Ilse Kryspin-Exner, Ruben C Gur, Ewald Moser

**Affiliations:** 1 MR Centre of Excellence, Medical University of Vienna, Lazarettgasse 14, 1090 Vienna, Austria; 2 Institute for Clinical, Biological and Differential Psychology, Faculty of Psychology, University of Vienna, Liebiggasse 5, 1010 Vienna, Austria; 3 Department of Psychiatry and Psychotherapy, University of Aachen, Pauwelsstrasse 30, 52074 Aachen, Germany; 4 Centre for Biomedical Engineering and Physics, Medical University of Vienna, Währingerstrasse 18, 1090 Vienna, Austria; 5 Center of Mind/Brain Sciences, University of Trento, Via delle Regole 101, 38060 Mattarello, Italy; 6 Department of Psychiatry, University of Pennsylvania Medical School, 3100 Spruce Street, Philadelphia, USA

## Abstract

**Background:**

The ability to recognize emotions in facial expressions relies on an extensive neural network with the amygdala as the key node as has typically been demonstrated for the processing of fearful stimuli. A sufficient characterization of the factors influencing and modulating amygdala function, however, has not been reached now. Due to lacking or diverging results on its involvement in recognizing all or only certain negative emotions, the influence of gender or ethnicity is still under debate.

This high-resolution fMRI study addresses some of the relevant parameters, such as emotional valence, gender and poser ethnicity on amygdala activation during facial emotion recognition in 50 Caucasian subjects. Stimuli were color photographs of emotional Caucasian and African American faces.

**Results:**

Bilateral amygdala activation was obtained to all emotional expressions (anger, disgust, fear, happy, and sad) and neutral faces across all subjects. However, only in males a significant correlation of amygdala activation and behavioral response to fearful stimuli was observed, indicating higher amygdala responses with better fear recognition, thus pointing to subtle gender differences. No significant influence of poser ethnicity on amygdala activation occurred, but analysis of recognition accuracy revealed a significant impact of poser ethnicity that was emotion-dependent.

**Conclusion:**

Applying high-resolution fMRI while subjects were performing an explicit emotion recognition task revealed bilateral amygdala activation to all emotions presented and neutral expressions. This mechanism seems to operate similarly in healthy females and males and for both in-group and out-group ethnicities. Our results support the assumption that an intact amygdala response is fundamental in the processing of these salient stimuli due to its relevance detecting function.

## Background

Functional neuroimaging studies focusing on the association of human amygdala with emotions, especially of its role in facial emotion processing in healthy subjects, have revealed activity related to several emotions, albeit with considerable inconsistence. For instance, Morris and colleagues [1,2] observed only left amygdala response when comparing emotional (happy, fearful) with neutral faces and enhanced left amygdala for fearful faces compared to happy. Likewise Blair and colleagues [3] reported left amygdala activation to sad expressions but no amygdala response to angry faces, whilst Nomura et al. [4] observed more pronounced right amygdala activation to subliminally presented angry faces than to neutral faces. Whalen et al. [5] reported amygdala activation to fearful and angry facial expressions. Divergent results on laterality of amygdala response and emotional valence also are reported by Britton et al. [6] and influence of stimulus material on amygdala activation has been demonstrated by Hariri et al. [7] who observed stronger right sided amygdala activation for emotional expressions (angry or afraid), whereas emotional scenes elicited stronger left-sided amygdala response. Only some studies have observed amygdala response to positive emotions (happy) [e.g., [8-12] right only]. The same holds true for the characterization of a response to all basic emotions presented [[13-16] left amygdala only] and only Chiao et al. [13] used an explicit emotion recognition task. Additionally, Sommer and colleagues [17] reported stronger left-sided amygdala activation during an emotional attribution task, supporting previous findings that proposed high involvement of the amygdala also in social judgments [e.g., [18,19]].

In sum, these results point to amygdala involvement in more neural pathways than just those responsible for fearful or threat related expressions, albeit with findings being extremely heterogeneous both in the emotions to which response is demonstrated and in laterality of activation. Given the technical difficulty of amygdala fMRI and the range of experiment designs employed, it seems likely that a number of methodological factors are responsible for the disparities between these studies. The anterior ventral brain region, where the amygdala is located, is affected by susceptibility artifacts especially in low-resolution measurements and at high magnetic fields. This can lead to low signal-to-noise ratio (SNR), and may account for failures to observe amygdala activation in single subjects [20,21].

Most studies have examined female and male subjects without analyzing the influence of gender, e.g. [13,16,17], despite its demonstrated importance in affecting neural systems involved in emotion processing (for reviews see [22,23]). Furthermore, the ethnic group of poser has been shown to be an influencing factor on the response of the amygdala [24-27], mostly indicating stronger reactions to out-group faces. However, these studies also postulated several modulating factors for this poser effect on amygdala activation, such as presentation time or task instruction. A critical point probably accounting for some of the reported divergences may be the definition of the emotional function or in other words the applied task: most studies used passive viewing and implicit emotion processing tasks, requiring for instance simple gender discrimination, e.g. [2,3,28], without an adequate discussion on the definition of the target function and the limitations in possible interpretations and generalizations. Often these tasks have implicitly been equated to emotion recognition though they are not really comparable in task requirements and processing load, since explicit emotion recognition tasks may require rather different neural networks than passive viewing or matching tasks. Moreover, there is no consensus on a general definition of emotion recognition, emotion processing, and emotion discrimination, which differ in their functional substrates but are experimentally not clearly separated from each other. For example, emotion matching paradigms [7] are also referred to as measuring emotion recognition, however, subjects are never asked to explicitly recognize or classify the displayed emotion. Thus, those paradigms lack information if subjects really knew which emotion they actually matched, if these were two happy faces, two angry faces or two fearful faces and any confusion of emotions cannot be validated. Despite the fact that emotion recognition is a function of major relevance and clinical impact, explicit emotion identification tasks have rarely been applied in studies employing neuroimaging tools but possess the opportunity to correlate behavioral performance (recognition accuracy and reaction time) with neural correlates providing new insights in the relation between neural activation and behavior.

In a preceding study from our lab optimized fMRI methodology was applied to examine the impact of task instruction on amygdala activation by directly comparing implicit vs. explicit tasks (implicit: age discrimination vs. explicit: emotion recognition; both using facial expressions from the same stimulus set [29]). There, the relevance of the amygdala during emotional face processing was highlighted, however, stronger activation occurred during explicit emotion recognition. To further characterize the role of the amygdala during these explicit emotion recognition demands with respect to different emotion qualities one objective of the present study was that to directly compare between the single basic emotions applied. This means that we extended previous studies by not only performing the simple comparison between positively and negatively valenced stimuli as shown before [e.g., [8]]. Moreover, we aimed at further exploring the impact of gender of rater and ethnic group of poser on amygdala activation, as previous studies, mentioned above, reported inconsistencies or did not explicitly address these issues.

Based on previous results [16,29] and regarding its evaluation and relevance detection function [30] we tested the hypothesis that all emotional and neutral facial expressions elicit bilateral responses of the amygdala when the task requires the explicit verbal categorization of an emotional facial expression. Therefore, we selected a large sample of Caucasian females and males and measured high-field BOLD changes in response to an explicit facial emotion recognition task applying advanced measurement and analysis techniques optimized for ventral brain regions [31-33]. The explicit emotion recognition task consisted of emotional expressions (anger, disgust, fear, happy, and sad) and neutral faces and participants were asked to choose the correct emotion from two possibilities presented on the left and right of the image as accurately and as quickly as possible, by pressing the corresponding button of a response box using the right hand (a more detailed description of the task can be found in the Materials and Methods section and elsewhere [34]).

## Results

### Behavioral data

The behavioral data of 3 female subjects (out of 25 in total) could not be included in the analysis due to a technical problem with the response device. For the emotion recognition performance during scanning the repeated measures ANOVA revealed a significant emotion effect (F(5,230) = 3.813, p = 0.006), no significant effect of poser ethnicity (F(1,45) = 1.200, p = 0.279), a significant interaction between emotion and poser ethnicity (F(5,230) = 22.980, p < 0.001) but no significant gender effect (F(1,45) = 0.116, p = 0.96). Additionally, neither a significant poser ethnicity-by-gender interaction (F(1,45) = 0.741, p = 0.394) nor a significant emotion-by-poser ethnicity-by-gender interaction (F(5,230) = 1.035, p = 0.392) emerged.

Considering the statistically significant interaction between emotion and poser ethnicity, post hoc tests revealed that angry (t = -2.959, p = 0.013), disgusted (t = -7.047, p < 0.001), and neutral expressions (t = -2.962, p = 0.005) were significantly better recognized in AA faces than in CA faces. For fearful (t = 6.189, p < 0.001), sad (t = 2.549, p = 0.014) and happy faces (t = 4.071, p < 0.001) recognition accuracy was higher in CA faces than in AA.

**Figure 1:**
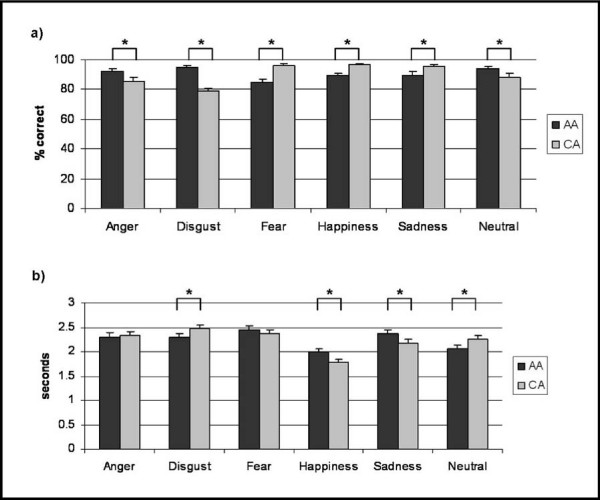
**Emotion recognition accuracy (a) and reaction times (b) for all emotions for African American (AA) and Caucasian (CA) faces**. Significant differences (p < 0.05) are marked by an asterisk.

Similarly for reaction times a significant emotion effect (F(5,41) = 24.122, p < 0.001) and a significant emotion-by-poser ethnicity interaction (F(5,41) = 8.568, p < 0.001) emerged, without significant gender effect (F(1,45) = 1.439, p = 0.237) or poser ethnicity effect (F(1,45) = 0.164, p = 0.688). The interactions between emotion and gender (F(5,41) = 1.078, p = 0.370) and between poser ethnicity and gender (F(1,45) = 1.448, p = 0.235) remained not significant. Post-hoc tests for the emotion-by-poser ethnicity interaction revealed that there was no significant difference in the reaction times for angry faces (t = 0.624, p = 0.536). However, reaction times were significantly shorter for AA disgusted faces (t = 2.533, p = 0.015) and neutral expressions (t = 2.919, p = 0.005) compared to the corresponding reaction times for CA faces. A trend for faster responses to fearful faces (t = -1.973, p = 0.054) was also notable. By contrast, sad (t = -3.251, p = 0.002) and happy faces (t = -3.791, p < 0.001) were recognized faster in CA faces. Identification accuracy and mean reaction times for all emotions across all subjects are displayed in Figure 1 and significant differences are marked.

Correlation analysis of recognition accuracy for each emotion with the corresponding response times revealed several significant relations, except for anger (r = -0.280, p = 0.056) which only reveals a trend, all indicating that the better the performance the faster the response (fear: r = -0.427, p = 0.003; disgust: r = -0.405, p = 0.005; happiness: r = -0.347, p = 0.017; sadness: r = -0.322, p = 0.027; neutral: r = -0.581, p < 0.001) or vice versa.

### Functional data

#### Whole slab analysis – Emotions

**Figure 2:**
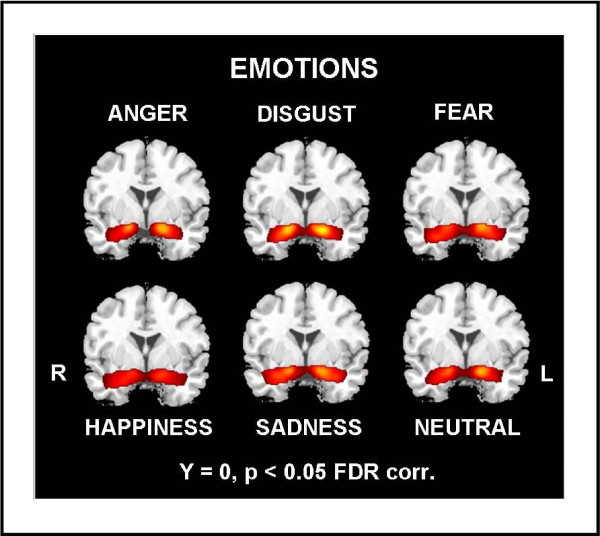
**Results of whole slab random effects analysis separately for each emotion across all subjects on one coronal slice (y = 0) passing through the amygdala (p < 0.05, FDR corrected)**. Analysis revealed bilateral amygdala response to all emotions and neutral expressions across female and male subjects.

For analysis of emotion effects we pooled data over both sexes as well as over CA and AA faces and assessed the differential neural response to the various emotional categories. All emotions as well as neutral expressions provoked a significant response of the amygdala bilaterally (see Figure 2 and Table 1).

**Table 1:** Results from random effects analysis showing Z-values, cluster size and p-values for left and right amygdala regions (MNI x,y,z: +/- 20, 0, -20, small volume corrected) for all emotions and neutral expressions separately.

**Contrast**	**L/R**	**MNI-Coordinates**			**Z-Values**	**Cluster-size**	**p-Value**
		**X**	**Y**	**Z**			
	R	16	0	-14	6.45	394	
**Anger**	L	-18	0	-16	6.74	416	

	R	16	2	-18	7.01	445	
**Disgust**	L	-20	0	-18	7.34	443	

	R	16	-4	-12	5.57	458	
**Fear**	L	-18	0	-12	6.59	437	p < 0.001 FDR

	R	12	-2	-16	4.96	459	
**Happiness**	L	-18	2	-18	5.21	446	

	R	16	0	-16	6.29	447	
**Sadness**	L	-18	-2	-16	6.46	424	

	R	18	2	-18	5.90	440	
**Neutral**	L	-18	0	-18	6.61	438	

Results of an ANOVA with emotion as within-subjects factor revealed no significant difference between emotions and neutral expressions in the amygdala activation (p < 0.05 FDR).

Correlation analysis of recognition accuracy for each emotion presented during fMRI and corresponding BOLD signal revealed a significant moderate positive correlation (left: r = 0.39, p = 0.007; right: r = 0.42, p = 0.004) between accuracy and amygdala response to fearful faces only. No further correlation of recognition accuracy or reaction time with BOLD response in the amygdala region reached significance after correction for multiple correlations (all p's > 0.008).

Interestingly, an exploratory calculation of correlation coefficients for females and males separately revealed a significant association between recognition accuracy and amygdala activation to fearful faces only in the male group (left: r = 0.53, p = 0.006; right: r = 0.61, p = 0.001; females: left: r = 0.33, p = 0.127; right: r = 0.26, p = 0.249). Applying a Fisher Z transformation to control whether correlations differ significantly from each other revealed no significant result (left: Z = 0.823; right: Z = 1.473, both Z < 1.65 which is the critical Z-value for alpha = 0.05). For reaction time and amygdala activation to fearful faces no correlation reached significance in both groups (females: left: r = -0.23, p = 0.304; right: r = -0.33, p = 0.129; males: left: r = -0.39, p = 0.055; right: r = -0.36, p = 0.079) though a trend in the male sample was notable.

#### Poser ethnicity

Examining the influence of ethnic group of poser we observed bilateral amygdala response to all emotional and neutral expressions of AA and CA actors (p < 0.05 FDR corrected). Direct comparison between activation maps of AA and CA faces for the different emotional expressions and neutral condition showed no significant difference in amygdala activation for any emotion (p < 0.05 FDR corrected).

#### Gender of rater

Females and males showed bilateral amygdala activation to every emotional condition (p < 0.05 FDR corrected). Direct comparison between activation maps of females and males for each emotion revealed no significant difference in amygdala response (p < 0.05 FDR corrected).

In the whole slab analysis we observed neural responses of other brain structures besides the amygdala to the facial stimuli, in particular fusiform gyri, inferior temporal and frontal regions. As the focus of this work was to characterize the association of the amygdala with expressions of basic emotions and facing the fact that the focus on 10 slices around the amygdala restricted analysis to ventral brain structures we present activation results only for the amygdala region.

#### ROI analysis

**Figure 3:**
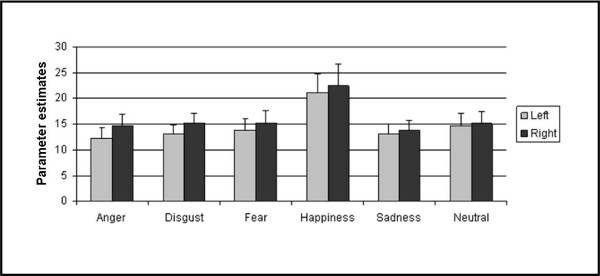
**Results from ROI analysis: mean parameter estimates (with S.E.M) for left and right amygdala for each emotion across all subjects**. Repeated measures ANOVA revealed no significant emotion effect (p = 0.083), neither a poser ethnicity effect (p = 0.398), nor any lateralization (p = 0.078) or gender effects (p = 0.613). Moreover, no interaction reached significance.

Results of the ROI analysis for the amygdala region revealed no significant emotion effect (F(5,240) = 2.335, p = 0.083), no significant effect of poser ethnicity (F(1,48) = 0.728, p = 0.398), no laterality effect (F(1,48) = 3.240, p = 0.078), and no significant gender effect (F(1,48) = 0.259, p = 0.613). In addition, no interaction reached significance (all p's > 0.281). Thus, results of the ROI approach fully support the findings from the whole-slab analysis. See Figure 3 for mean parameter estimates.

## Discussion

### Emotions

Our results demonstrate strong bilateral amygdala activation during the explicit identification of *all *applied basic emotions and neutral facial expressions, never shown before in studies addressing emotion identification. These findings support the hypothesis that all facial expressions of emotions – irrespective of valence – would elicit amygdala activation when there is an explicit requirement to identify the emotion. Together with previous results demonstrating amygdala activation during incidental emotional face processing [29,35], our data support the theory that the amygdala serves an appraisal role and prioritizes a fast and raw evaluation mechanism for emotionally salient cues [36-38]. Robust amygdala responses occurred also during happy and neutral face processing, consolidating the conclusion that responses of the amygdala are not limited to the processing of negative stimuli. Sander, Grafman and Zalla [28] considered the amygdala as a 'relevance detector' which not only explains responsiveness to fearful and negative stimuli but also to positive and even neutral stimuli, hence a broader spectrum of biologically relevant stimuli, which finds support in animal models [e.g., [39,40]] and human studies across different modalities (static faces: e.g., [8,14-16]; auditory stimuli: e.g., [41,42]; olfactory stimuli: e.g., [43]; moving whole-body cues: e.g., [44]; for review see [45]). This is in accordance with results from studies on backward masking, e.g. [46], suggesting some kind of monitoring function of the amygdala, i.e. we never ignore a facial expression as it might be of relevance, further strengthening the assumption of the evaluative function of the amygdala, assessing the behavioral relevance associated with a specific socio-emotional stimulus and initiating the adequate responses (approach/avoidance). The same phenomenon – amygdala activation to neutral stimuli – has been perfectly demonstrated in LeDoux's famous figure [36] depicting the subcortical and cortical pathways toward the amygdala and the differentiation between an obviously neutral stick that might be misjudged as a snake in the first appraisal when only information of the subcortical pathway is processed. In our study we did not see a significant difference in amygdala activation between the emotions, however, even with optimized protocol and increased sensitivity fMRI today might not be able to map qualitative differences due to inherent restrictions in temporal and spatial resolution.

The fact that we observed strong amygdala activation to happy expressions might be linked with the fact that happy represented the only positive emotion hence novelty effects [cf. [47]] might have triggered stronger reactions as all happy faces are characterized by a distinct expression and clearly differ from all negative emotional expressions that show more overlap in expression.

Notably, a positive correlation emerged between BOLD signal change in the amygdala and recognition accuracy, and a corresponding negative correlation with response times, for fearful faces only. Furthermore, correlation analysis of recognition accuracy and reaction time revealed a significant negative relation indicating that subjects who took longer made more errors. In light of the significant correlations of behavioral parameters with BOLD signal in the amygdala for fearful faces one can assume that the higher the amygdala activation the better the performance and the faster the response.

To further treat this discussion, the elevated amygdala activation might also be influenced by emotional contagion as has been shown by several previous studies, e.g. [48,49], indicating that emotional contagion and simulation of fearful faces might prompt stronger amygdala activation than the simulation of other emotional expressions, that are less threatening.

The positive correlation between recognition accuracy and BOLD signal in the amygdala supports our preceding results [29] were we found a positive correlation between amygdala response and emotion recognition performance, but not age discrimination. However, only for fearful expressions we observed a significant impact of behavioral performance on amygdala activation, which does not occur for other emotions or neutral expressions though no ceiling effects for the other emotions occurred. This suggests a strong affinity between fear and amygdala probably driven by ambiguity of stimuli as described by Whalen [50]. Hence, our data implicate that the amygdala's role is to monitor and evaluate all human facial expressions though a direct influence on behavior seems to emerge only for fear processing. Notwithstanding the universal response of the amygdala in processing all facial expressions, our results may underline the special role of fear within the basic emotions as the response to fear has a unique contribution to survival, providing a signal for danger, and hence has a special relevance among other emotions, requiring both a fast and correct responding. Consequently, most previous studies on emotions reported amygdala activation to fearful faces, e.g. [1,2,5,8,13,15-17,51].

### Lateralization

We found bilateral amygdala activation for every facial condition used and observed no significant lateralization effects, either in the whole slab or in the ROI analysis. Both analyses revealed similar results, underlining the application of an optimized protocol when measuring ventral brain activity. Hence, our data support preceding results on bilateral activation during emotion processing [29] and meta-analyses of neuroimaging studies demonstrating no consistent lateralization effect [52,53]. Hartje [54] suggested a general dominance of posterior regions of the right hemisphere for emotion processing. Right amygdala response has been associated with sensory driven, bottom up processing [55] and processing of ambiguous stimuli, whereas left amygdala activation is rather implicated in more cognitive appraisals of emotional stimuli [56,57]. Left lateralization of amygdala activation has been frequently reported for emotion processing tasks, e.g. [8,58-60], and recently Fitzgerald and colleagues [16] reported left only amygdala activation in processing of emotional faces. Our findings of bilateral amygdala activation are in line with results from interaction analysis [61] and functional connectivity analyses of regions involved in emotion processing [61,62]. Accordingly, we assume that lateralization effects are subject to temporal dynamics [63] and task requirements, e.g. emotional memory [64,65].

In previous studies increased signal drop out in this region has been reported potentially leading to lateralized activation as a result from unilateral low SNR in the amygdala of some subjects [20,21]. Using our methodological improvements (i.e., optimized high resolution EPI protocol as well as physiological artifact correction) has led to reduced signal drop out in this brain region and likely – together with the applied task – explains the absence of findings of lateralization. Preceding results from another group of 29 healthy subjects also failed to show a lateralization effect [29].

### Poser ethnicity

We observed amygdala activation during all conditions for both ethnic groups, further supporting the general evaluative function of the amygdala for socially relevant cues irrespective of cultural background. Previous research on ethnic group has demonstrated stronger amygdala response to out-group faces [27,59,66], stimulating discussion and further studies on amygdala function in ethnic biases [13,24,25], prejudicial attitude [26,67], social judgments and evaluation processes (for review see also [68]). Results from amygdala lesion studies have demonstrated that social judgments such as assessing trustworthiness or approachability by perceiving the face are strongly impaired [18]. Previous indication for amygdala involvement in more subtle social judgments was provided by an fMRI study by Winston and colleagues [19], reporting stronger amygdala activation to faces rated as untrustworthy. Thus, the amygdala's role appears to be of particular importance in retrieving socially relevant knowledge on the basis of facial appearance. In the behavioral data, significant influence of ethnic group on performance was apparent, although not as would be predicted from the in-group-advantage hypothesis [69]. Indeed, recognition accuracy was better and response times shorter for AA expressions of anger, disgust and neutral in this entirely Caucasian sample. In contrast, expressions of happiness, sadness and fear were more accurately and faster recognized in CA faces. However, we did not observe any influence of poser ethnicity on amygdala activation and thus our data only partly support recent findings by Chiao et al. [13] who also investigated explicit emotion recognition in Japanese and European American subjects using a very similar task with Japanese and European American faces. They observed significantly stronger amygdala activation to fearful in-group faces in both cultural groups, but no impact of ethnic group of poser for the other emotions. We failed to show any poser ethnicity effect on the neural level of the amygdala but observed a significant difference in the behavioral data, where fearful in-group faces were recognized more accurately. Despite the fact that our sample consisted of only one ethnic group (Caucasian Austrians), we studied twice as many subjects than Chiao et al. and applied an optimized fMRI protocol that was especially sensitive for amygdala activation. Moreover, stereotypes associated with Asian vs. AA faces might differ, in particular regarding perceived threat and increased aggressiveness, e.g. [59], probably also accounting for differences in the observed activation patterns.

The amygdala is but one structure in the neural circuit underlying recognition of emotions and possesses several cortical and subcortical feedback routes. Influences of ethnic group of poser might also be processed in other regions known to be essential for identifying emotions (e.g., insula, fusiform gyrus, orbitofrontal cortex, anterior cingulate and lateral prefrontal cortex), which have not fully been covered by our protocol thus allowing only restricted analysis. Furthermore, the better performance for in-group expressions of happiness and sadness, emotions that have a strong social aspect, could possibly be driven by stronger empathy for in-group faces. Higher accuracy for out-group expressions of disgust and anger, two negative and complex emotions in part indicating avoidance, might be especially important when expressed by out-group posers thus eliciting more attention. To further characterize these emotion dependent performance differences, the frequency and quality of contact with members of other ethnic groups, as well as sympathy ratings for the presented faces, should be considered as these might modulate neural activation and performance. Moreover, Rozin, Lowery and Ebert [70] pointed out that posed expressions of disgust containing nose wrinkle and the combination of gape and tongue extension are most clearly associated with negative sensory events and oral irritation – the "core disgust". However, authors also discuss the upper lip retraction as a sign for "extended disgust", which might be stronger influenced by cultural background and also harder to recognize. Despite the fact that all facial expressions have been validated before and showed recognition accuracy above 70% correct, our evoked Caucasian expressions of disgust clearly differed in expression style with half of the stimuli showing upper lip retraction and the other half with gape and tongue. Regarding the African-American stimuli, two third of the pictures comprised expressions of "core disgust", probably enabling higher recognition accuracy.

Concerning the emotion recognition data obtained from the VERT-K, an exploratory confusion analysis indicated that CA expressions of disgust were most frequently mistaken as sadness (22% of all answers to disgust faces) followed by the confusion of sadness with disgust (17%). Previous data indicate a stronger confusion of disgust with anger but also with sadness [cf. [71]] which might be due to various factors, e.g., expression style (core vs. extended disgust) and also intensity of expressions. Irrespective of poser ethnicity and answering format, disgust is a complex emotion and its correct recognition seems to be modulated by several factors.

### Gender of rater

Both female and male participants exhibited robust bilateral amygdala activation to all presented emotional and neutral expressions and no significant gender difference in amygdala activation was observed. This is in accordance to previous reports of bilateral amygdala activation to fearful faces in both genders [51].

Furthermore, we observed a significant correlation between recognition accuracy and amygdala activation to fearful stimuli across all subjects that – interestingly – was driven mainly by the male sample. Separate correlation analyses for females and males revealed a significant relationship only for the latter group for both, the left and right amygdala. Hence, our data indicate that gender differences might only occur on a subtle basis – in our study affecting the association between behavioral responses and amygdala activation, while neither behavioral nor neural responses alone revealed gender differences. Thus, our data are in perfect concordance with results from Schneider et al. [72] who also observed a significant correlation between mood parameters and amygdala activation during sad mood induction only in the male subjects, while no significant gender difference in mood parameters occurred. The assumption that gender may exert its influence on a relatively subtle basis might be supported by previous studies that frequently reported lateralization differences in amygdala activation between females and males thereby lacking any gender differences in behavioral measures or general amount/extent of amygdala activation [73-75]. These slight differences could reflect different cerebral strategies of emotion processing resulting in a similar behavioral or functional outcome. The reason for such different strategies remains however unclear and should be investigated in greater detail in future studies.

### Limitations

To gain stronger signal change and avoid susceptibility-based signal loss and movement artifacts in the ventral brain regions we used an event-related paradigm and could acquire only 10 slices, thus accepting restricted brain coverage. This enabled us to examine the amygdala with optimized methodology; still a differentiation of activation peaks into the separate nuclei seems not reasonable. This should be pursuit in future studies since previous research indicated that the basolateral nucleus possess the majority of afferences and is known to be important for learning [76]. The central nucleus is the principal output nucleus for the expression of conditioned fear responses and is essential for adaptation to chronic stress, e.g. [77]. It might be relevant for a wide range of attentional processes as has been shown in animal studies, including both those involved in the acquisition of new information and those involved in directing action, e.g. [78]. Moreover, in light of recent findings of modulation of amygdala activation by hormone concentration during emotion processing, involvement of this particular region should be further explored. Moreover, for most other regions known to be involved in emotion processing whole brain analysis would be required to study the complex neural circuits of facial emotion recognition, implicating an interactive network with distributed activity in time and space (for review see [79]). Furthermore, the impact of personality factors as well as hormone levels on neural activation was not examined, but would definitely help to further characterize the function and pattern of the emotional circuitry.

We applied an explicit emotion recognition task showing in-group and out-group faces which were accompanied by two emotional words (one on the left and right side of the face), to specifically assess emotion recognition performance. As we intended to examine the role of the amygdala during identification of emotions in facial expressions we relied on this special task. Here, even a matching task (using one emotional face as target and two alternative faces as responses, one depicting the same emotion) is not adequate, since it does not require the explicit categorization of the face but rather a decision based on similarity. It could therefore happen that the subject is not able to name the emotion but nevertheless finds the correct emotional face corresponding to the target. Thus, passive viewing tasks, gender discrimination tasks or matching tasks using emotional expressions may involve some kind of emotion processing they, however, do not adequately tap the socially highly relevant function of emotion discrimination.

Despite the problems associated with emotional words as response categories we are not aware of other practical ways to examine the neural correlates of this basic social ability. To our opinion response devices with more than two or three response possibilities are not feasible in fMRI settings and would hold too many sources of error since subjects have no visual control of their responses potentially leading to confounds. Furthermore it introduces other confounding factors such as cognitive processes in form of a higher working memory load.

Despite the listed advantages of explicit emotion recognition tasks and the justification for using it, the answering format and the small amount of stimuli per subgroup prevented careful analysis of error tendencies, which would have been useful for interpreting the in-group/out-group performance differences for some emotions. Hence, future studies investigating emotion recognition should apply more elegant designs that even allow analysis of error tendencies.

The combination of behavioral with neural results revealed a significant correlation between fear recognition accuracy and amygdala activation supporting the special relationship between this particular emotion and an intact amygdala response. In addition, further analyses and clarification, e.g., via analysis of incorrect responses, are needed but are lacking in the current study due to the limited amount of errors in this healthy sample.

## Conclusion

We observed bilateral amygdala activation towards all emotions during an explicit emotion recognition task and consider this a substantial evidence in the literature on emotion processing and amygdala activation since results rely on a large sample size, are qualitatively optimized with respect to the amygdala and clarify the impact of different important influencing factors, such as gender, culture and emotion on explicit emotion recognition. Hence, the robust, bilateral amygdala activation in response to all emotional expressions of faces of different cultural background indicates that an intact amygdala response as an important part of the underlying neural network seems to be fundamental for the evaluation of facial expressions of emotions. This mechanism seems to operate similarly in healthy females and males and for both in-group and out-group ethnicities. However, a significant correlation of amygdala activation and behavioral response to fearful stimuli occurred, in particular in the male sample. Hence, our data indicate that gender differences might only occur on a subtle basis – in our study affecting the association between behavioral responses and amygdala activation, while neither behavioral nor neural responses alone revealed gender differences. This indicates that the specific association between an intact amygdala reaction and the processing of fearful faces may not only be task dependent but also subject to gender influences.

## Methods

### Participants

Twenty-five right handed healthy Caucasian females aged 19–36 years (mean age 24.5 years, SD = 3.8) and 25 right handed healthy Caucasian males aged 20–35 years (mean age 25.6 years, SD = 3.9) participated in the study. They were recruited by advertisements posted at the University of Vienna and the Medical University of Vienna, Vienna, Austria. All subjects were paid for their participation and written informed consent was obtained. The study was approved by the local ethics committee and subjects were treated according to the Declaration of Helsinki regarding treatment of human research participants.

The presence of psychiatric disorders (according to DSM IV) was excluded on the basis of the German version of the Structured Clinical Interview for DSM (SCID) [80] conducted by experienced clinical psychologists. The usual exclusion criteria for MRI also applied. Females and males were of similar age (p = 0.347) and years of education (p = 0.851; females, mean = 17.44 years, SD = 3.0; males, mean = 17.28 years, SD = 2.9).

All subjects completed two questionnaires measuring handedness [81,82] and alexithymia (TAS) [20,83] and no gender difference for alexithymia appeared (p = 1.000; mean females: 48.28, SD = 6.6; mean males: 48.28, SD = 7.7).

### Functional tasks

**Figure 4:**
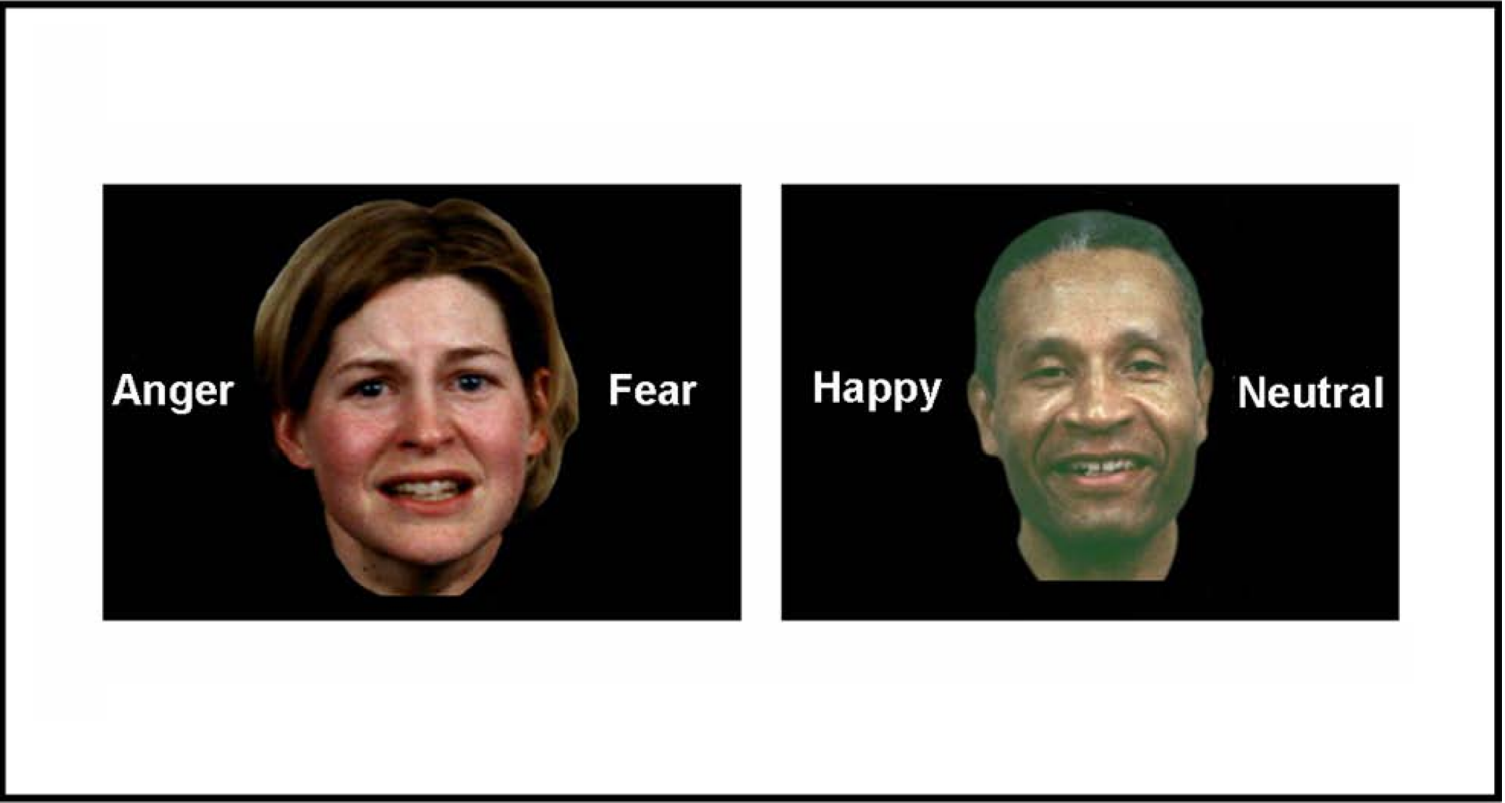
**Examples from the emotion recognition task**.

The functional task applied here has been described in more detail elsewhere [29,34,84,85]. Briefly, the stimulus material consisted of color photographs of facial expressions portraying an equal number of the five basic emotions (anger, disgust, fear, happiness and sadness) and neutral expressions. All expressions were taken from a stimulus set that has been standardized and used repeatedly as neurobehavioral probes in neuroimaging research [16,29,34,35,84-88]. The stimulus material applied here was also validated for the Austrian population [89]. The stimuli were balanced for emotion (12 per each emotion and 12 neutral) and gender (36 female, 36 male pictures). Each actor appeared only once. To investigate the influence of poser ethnicity, half of the stimuli were Caucasian (CA) faces; the other half African American (AA) and stimuli were equally distributed across facial expressions and gender such that for each emotion and neutral 3 stimuli were AA women, 3 were CA women, 3 were AA males, and 3 were CA males. Stimulus presentation was randomized with regard to emotion, poser ethnicity and gender, and the order of presentation kept constant between subjects. Subjects were instructed to choose the correct emotion from two possibilities presented on the left and right of the image as accurately and as quickly as possible, by pressing the corresponding button of a response box using the right hand. One of the options was correct and the other was selected at random from all other choices. Emotional facial expressions were presented for a maximum of 5 s with a randomized, variable interstimulus interval (ISI) ranging from 12 s to 18 s (during which subjects viewed a scrambled face with a central crosshair). Manual responses triggered immediate progression to the next ISI. Stimuli were projected onto a screen and viewed by the participants via a mirror mounted on the head coil. The presentation of images, recording of responses and acquisition of scanner triggers (one per TR) was achieved using the Presentation^© ^software package (Neurobehavioral Systems, Inc., Albany, CA). Figure 4 shows examples of emotion recognition task stimuli.

### Behavioral data analysis

The behavioral data (emotion recognition performance and reaction time) acquired during scanning were analyzed with repeated measures ANOVAs, with emotion (anger, disgust, fear, happiness, sadness and neutral), poser ethnicity (Caucasian and African American) and laterality as within-subjects factors and gender as the between-subjects factor. Greenhouse-Geisser corrected p-values were used for all ANOVAs and post hoc results were Bonferroni corrected.

### FMRI acquisition parameters and data processing

#### Data acquisition

We used the same echo-planar-imaging (EPI) protocol specifically optimized for measuring amygdala activation at 3 T [31-33], as has been described in recent studies by our group [29,34,84,85].

All subjects were examined with a 3 Tesla Medspec whole-body scanner (Bruker Biospin, Ettlingen, Germany) at the MR Centre of Excellence, Medical University of Vienna, Austria. Functional Imaging was performed in the axial plane using gradient-recalled EPI. Ten oblique axial slices centered on the amygdala were acquired using asymmetric k-space sampling (FOV = 25 × 21 cm, matrix size 128 × 91, slice thickness 2 mm, slice gap 0.5 mm, TR = 1000 ms, TE = 31 ms). Cardiac action and breathing were digitally recorded to allow physiological artifact correction in post-processing using physiofix [90] and retroicor [91] algorithms, which further increased sensitivity [92].

#### Data preprocessing

Functional data were preprocessed using SPM2 . Images were slice timing corrected, realigned to the mean image and normalized into the standardized stereotactic space. For reasons of physiological and thermal noise, to increase SNR and allow inferences as to statistical significance in the context of the Gaussian random field theory, functional data sets were spatially smoothed using an isotropic Gaussian kernel with a full-width-at-half-maximum of 9 mm; see also [93,94].

For this event-related design, each stimulus was modeled with a separate regressor, based on the individual response period convolved with the canonical hemodynamic response function and its temporal derivative. This enables the calculation of contrasts for each valence separated by ethnic group of poser. An additional regressor without hemodynamic delay was used to account for signal changes due to head motion during stimulus presentation. Regressors of each valence and group of poser were pooled to assess brain responses to each emotion by ethnic group of poser leaving a minimum of 6 stimuli per category for statistical analysis.

Statistical analysis was performed at the individual and group level. To detect group differences, contrast images from all subjects were included in a second level random effects analysis. Gender differences were calculated with two sample t tests. Effects of poser ethnicity were calculated with paired t-tests. Differences between the emotions were examined by applying a one-way ANOVA with emotion as within-subjects factor.

#### ROI analysis

To examine the implications of different analysis methods both whole-slab and region of interest (ROI) approaches were applied. Since our main hypothesis focused on the amygdala and the results of the voxel-wise whole brain analysis may be affected by minor mislocalization due to imperfect normalization, we performed an additional ROI analysis with the aim of maximizing the sensitivity to amygdala results. A further aim was to analyze possible hemispheric lateralization effects in greater detail. Note that the data sets were spatially smoothed by a Gaussian kernel of 9 mm, before whole-brain and ROI analysis, corresponding approximately to the size of the amygdala. ROI definition was based on the MRIcro amygdala template . Mean parameter estimates were extracted for left and right amygdala ROI in each condition and subject using IDL (Interactive Data Language, RSI Inc., Boulder, CO, USA). Levene tests for homogeneity of variances indicated homoscedasticity for all parameter estimates of all emotions (neutral: p = 0.142; anger: p = 0.248; disgust: p = 0.392; fear: p = 0.312; happy: p = 0.184; sad: p = 0.405), left and right amygdala (left: p = 0.334; right: p = 0.725) and ethnic group of poser (AA: p = 0.375; CA: p = 0.678). A four-way ANOVA was applied with gender as between-subjects factor and emotion, poser ethnicity and laterality as repeated factors. Greenhouse-Geisser corrected p-values are presented.

#### Corollary analyses

Correlation analysis was performed for each emotion between the behavioral parameters (recognition accuracy with reaction time), recognition accuracy (correct responses), and BOLD effect (Z-transformed values of the mean parameter estimates within the amygdala as shown by SPM random effects analysis), as well as reaction time (seconds) and BOLD effect. As variation in correct response rates were not distributed normally Spearman rank correlations were calculated. For correlations that reached significance across all subjects we further calculated separate correlation coefficients for female and male participants and significant differences between correlations were tested after Fisher Z transformation [95].

Results for the whole group, female and male subjects separately, and direct comparisons are presented at an FDR corrected threshold of p < 0.05.

## Authors' contributions

BD and UH designed the study, recruited subjects, analyzed behavioral and functional data, and wrote the manuscript. CW and SR were the physicists who conducted the fMRI measurements, helped with functional data analyses and writing of the manuscript. IKE, RCG and EM helped with interpretation of data and the final version of the manuscript. All authors contributed to and have approved the final manuscript.
